# After War Conditions

**Published:** 1919-11-01

**Authors:** 


### THE MATRONS' AND SISTERS' DEPARTMENT.

### AFTER WAR CONDITIONS.

### Producing Inconsequent Conduct and Speech.

The present state of affairs in the industrial world seems to have given hope for the doing of a new kind of business in an entirely novel field by certain types of persons who are out for making a living in fresh fields and pastures new. A terse account of a "mass meeting" in Mortimer Hall, W., on Saturday, October 25, admission to which was free, could not fail to amuse the sceptical. The members present were recruited by the issue of handbills and literature in those places where nurses are most frequently to be met with. Saturday afternoon was a favourable time for such a meeting, as it enabled all those who were in search of amusement or excitement to spend a little of their leisure in listening to speeches which, whatever their merits, caused much amusement to sections of the audience as the proceedings advanced. It was soon manifest that the Divider of the nursing world, the leader of the party which set out to deceive the House of Commons and failed, with a few inconsequent but noisy people who, for two decades of years at least, have endeavoured to make a name by exploiting nurses, whenever possible, by threats and imprecations, had in their unwelcome and consequent leisure decided to attempt to trouble once more for their own purposes the nurse who is dependent upon her earnings for her maintenance and security. Looking round the room, though a certain camouflage was adopted, the aforesaid disturbers soon became apparent, though their tactics at the moment led them to lie low. The gathering, though large, was of a mixed character, and the more vocative of those present manifested themselves as belonging to the unemployed and unsatisfied sections of the community with many who came to spend a pleasant Saturday afternoon. How many actual *bonâ fide* certificated nurses were present it would be difficult to say, but the promoters soon made it manifest that a large portion of the audience could not be placed in this category.

The quality of the speeches may be judged from the line taken by one of the most abusive and venemous of the speakers, whose idea was to set about destroying the paper which omitted to publish her communications. To this end, she informed her audience, she had boldly gone to the office and obtained two copies; she then stood on the doorstep outside, where, raising her hands to the heavens, she tore the publication into small pieces and scattered them on the pavement before the door. If all the nurses or any large number of them were to follow this example, the financial success of the publication in question might soon become greater than that of any publication circulating in the Metropolis. This fact will be grasped by the overwhelming body of nurses throughout the country, and they are not likely to support, or even countenance the propagation of nonsense of this description. The chair was occupied by a disappointed member of the Nurses' Co-operation. She was supported by Miss Macdonald of the Royal British Nurses' Association, who will probably be invited to give an explanation of her utterances, conduct and presence at such a gathering by Her Royal Highness, who is not likely to suffer without protest the besmirching through its chief official of the good name of the institution for which H.R.H. gained the Charter. A lawyer and an actor completed the quartet who faced the audience as occupants of the platform. Free speech was tabooed by the audience, only those who declared themselves to be warm supporters and advocates of a Nurses' Trade Union being allowed to utter a word. This Fenwick touch and intolerance demonstrated the consciousness of these would-be disturbers of the nurses' independence and peace to the impotence their continuous efforts in this direction have won for themselves. The camouflage adopted at Mortimer Hall recalled a former meeting more than twenty years ago, when what are now known as "Fenwicks" with certain nefarious tactics first became manifest. On that occasion they wanted a majority of votes, and set out to secure it at a certain meeting by collecting the kitchen and other maids, putting them into nurses' dresses, shepherding them to the Hall of Assembly to hold up their hands as representative members of "The Intelligentsia" (?) of the nursing body, of whom these ingenious agitators are the parents, rulers and sole exponents. Is it not time to end the residue of the old stage army and put them out of mischief? There is no room for such pretenders in the field in which the working nurse has to labour, move, and win her livelihood and reputation.

The kernel of the whole matter is that no highly qualified nurse can support a trade union at any price.

